# ‘The Microbiome and the Pathophysiology of Asthma’

**DOI:** 10.1186/s12931-016-0479-4

**Published:** 2016-12-05

**Authors:** Ashley Sullivan, Eoin Hunt, John MacSharry, Desmond M. Murphy

**Affiliations:** 1 The Department of Respiratory Medicine, Cork University Hospital, Wilton, Cork, Ireland; 2 APC Microbiome Institute, School of Medicine, University College Cork, Cork, Ireland; 3 School of Microbiology, University College Cork, Cork, Ireland; 4 Health Research Board Clinical Research Facility, University College Cork, Cork, Ireland

**Keywords:** Asthma, Host, Microbe dialogue, Translational research, Medicine, Inflammation, Microbiology, Epithelium, Hygiene hypothesis

## Abstract

Asthma is a chronic respiratory disease whose prevalence is increasing in the western world. Recently research has begun to focus on the role the microbiome plays in asthma pathogenesis in the hope of further understanding this respiratory disorder. Considered sterile until recently, the lungs have revealed themselves to contain a unique microbiota. A shift towards molecular methods for the quantification and sequencing of microbial DNA has revealed that the airways harbour a unique microbiota with apparent, reproducible differences present between healthy and diseased lungs. There is a hope that in classifying the microbial load of the asthmatic airway an insight may be afforded as to the possible role pulmonary microbes may have in propagating an asthmatic airway response. This could potentially pave the way for new therapeutic strategies for the treatment of chronic lung conditions such as asthma.

## Background

Asthma has seen a staggering rise in prevalence in recent years with more than 300 million people worldwide believed to be affected by this chronic respiratory disorder [1–3]. The burden of asthma is felt globally, however it is in developed countries that asthma has come to the fore as a health care concern [4] where the incidence rate of asthma and in particular adult onset asthma has risen so dramatically that this respiratory disorder has reached epidemic proportions [5]. The incidence of childhood asthma in the Western world has also reached worrying levels [6]. Recently research has begun to focus on the role microbes play in this chronic respiratory disorder. Thanks to advances in sequencing technologies it is now possible to dissect the role microbial composition and diversity can play in a multi-factorial disorder such as asthma. It is beginning to become clear that there are clear differences present between asthmatics and non-asthmatics and the microbial load they carry. Through gaining an insight into the unique microbiota of asthmatics much may be revealed, as has happened in gastrointestinal microbial research, which could undoubtedly aid in disease prognosis and treatment.

### Microbes and the airway

While research into respiratory diseases has rewarded us with pharmacological treatments such as inhaled corticosteroids, leukotriene receptor antagonists, β agonists and more recently targeted monoclonal therapies which have helped alleviate the burden of asthma it is only since ca. 2010 that research has confirmed that the environment of the lungs are not sterile as had previously been believed [7, 8]. Prior to this the prevailing dogma was that if bacteria were detected in the lower respiratory tract it was understood to represent an abnormal health state and that the healthy lung was sterile [9]. This belief arose mainly due to poor, ineffective microbial culturing techniques which were unable to replicate the lung habitat and culture the microbes from respiratory specimens [10]. However it is clear that although the lung contains a smaller bacterial burden than in the upper respiratory tract there is a distinct microbiota present in the lower airways of healthy individuals [11]. This finding that the lungs harbour a unique microbiota irrespective of health or disease has led to the surge of research now taking place in an effort to not only categorise the distinct microbiota of the diseased lung but also that of the healthy lung [12]. Thanks to the advancement of culture independent techniques the unique steady state microbiota of the lungs is being elucidated [9]. A change in the microbial composition of the lower airway microbiota may be connected with chronic lung disease such as asthma [2, 13].

The development of inexpensive next generation sequencing techniques, developed for the Human Genome Project to sequence the human genome, has made it possible to determine microbial, in particular bacterial, communities in a culture independent manner. By screening the diversity of bacterial ribosomal rRNA genes present in biological nucleic acid samples against sequence databases and development of bioinformatic algorithms have enabled the organisation and interpretation of the huge data sets made available by these sequencing machines [14, 15]. Meta-genomic approaches have enabled a far greater understanding of the microbes present at various sites and the role they play in the overall host-microbiome interaction [15]. Thanks to culture independent techniques low levels of oral bacteria such as *Prevotella* and *Veillonella* have been discovered in the lower airways of healthy individuals [15–17].

### Lung environment

**Fig. 1:**
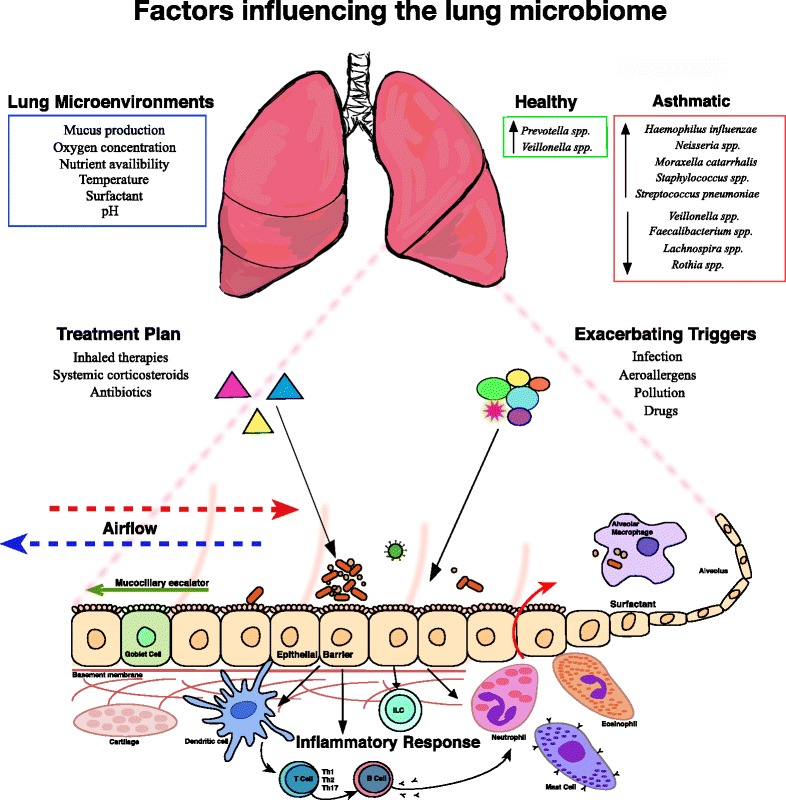
Factors influencing the lung microbiome. Schematic illustrating the complex factors affecting the lung microbiome in Asthma. The lung microenvironment selects for specific microbes which change in Asthma due to environmental factors, infection and treatment options which modulate the inflammatory cascade at the epithelial interface and the corresponding microbiome. Asthmatics display a changed microbiome with increased lung microbes such as *Haemophilus*, *Neisseria*, *Moraxella*, *Staphylococcus*, and *Streptoccoccus* with reduced *Veillonella*, *Faecalibacterium*, *Lachospira* and *Rothia* sp

With a surface area of 75 m^2^ and direct exposure to the environment, the lungs are prime sites for microbial exposure [10]. As the environmental conditions shape the composition of the microbial communities in that habitat it is important to understand the environment of the lungs to fully understand the potential microbes which could inhabit this location, see Fig. 1. The lungs cannot be viewed as one standard habitat, as they in fact consist of microenvironments in which differences in temperature, pH, mucus production, epithelial specialisation, the motility of the mucociliary escalator, oxygen concentration and nutrient availability all have the potential to influence the variety and type of microorganism present [18, 19]. Such physiochemical property gradients may plausibly allow for the survival of different microorganisms within varying regions of the airways. It should be noted however that a recent study has concluded that in the lungs of healthy individuals spatial variation in the microbiota appears to be far less significant than the variation present across individuals [20]. The authors also reported that the microbiota of the lung in health seems to be more influenced by the immigration and elimination of microbes rather than by the growth conditions influencing bacterial growth rates. Furthermore, this pattern may be reversed in patients with advanced lung disease due to alterations in the pulmonary environment induced by disease progression [20]. As the lungs are connected directly to the microbial rich oral and nasal passages, the potential for regular translocation of microbes from these sites secondary to inspiration and with possible associated micro aspiration, it is therefore not surprising that there may be similarities between the microbial composition of these sites. To investigate this further, Venkataraman et al.*,* utilised a neutral model for community ecology to differentiate members of the lung microbiome whose presence in the lungs is consistent with dispersal from other body sites and the members of the lung microbiome who deviate from this model, suggesting a competitive advantage for these microbes in the lung [21]. From this study dispersal of microbes from the oral cavity would seem to be the main mechanism whereby the lungs become colonised in healthy individuals and as no species constantly deviated from the neutral model one might postulate that it is the constant dispersal that is important for the microbial structure of the lower lungs rather than selective pressure [21]. However, the process could possibly be more complex than suggested in this neutral model, with the possibility that microbes introduced into the airways are killed at the same rate in which they are introduced, creating a homeostatic balance and therefore the perception of a neutral distribution, when in fact the lung environment is highly selective.

### Sample collection and the lungs

Due to the belief that the lungs were sterile and due to the invasive nature of bronchoscope sampling the lower respiratory tract was not, at first, included in the Human Microbiome Project [22]. As recent characterisation of the lung microbiota has revealed, there are definable reproducible differences between healthy and diseased state lungs. It is now accepted that the healthy lung microbiome is diverse and some studies have even shown that the respiratory tract can more rapidly acquire diversity after birth than the intestines [23]. While there is now acceptance that there are microbes present within the airway, the challenge remains in overcoming the difficulty in sampling and culturing these microbes from the lower respiratory tract. The validity of sampling techniques such as bronchoalveolar lavage (BAL) fluid and bronchial brushes have been subject to debate due to the potential for contamination from the oropharynx during bronchoscopy. Despite such concerns, recent studies have shown that, by comparing the microbial species present in the upper respiratory tract with the microbiota of the lower respiratory tract, the microbiota within the lower respiratory tract are unique. A recent study by Bassis et al., compared samples from healthy subjects collected by oral wash, BAL fluid, nasal swab and gastric aspirates and demonstrated that the oral microbiome did not contaminate BAL samples [22]. The oral cavity and stomach contained high species richness and the highest bacterial signal levels in comparison to both the nasal cavity and lungs which had much lower bacterial signal levels. Whilst it was seen that the bacterial communities present in the healthy lung overlapped with those present in the oropharynx, the concentrations were much lower in the lungs and there was a different community composition in samples from healthy lungs [22].

While the sampling technique is of concern with regards to contamination, caution should also be taken with sample processing and the generation and analysis of complex datasets. Unintentional environmental contamination when processing can lead to the unintentional identification of contaminants by the powerful sequencing technologies and controlling potential sources of contamination such as reagents is vital. It is also important to avoid bias and extraction protocols are critical. In sequencing of samples it is usually one of the variable regions of the 16S rRNA gene that is targeted by primers, however this is not ideal for the detection of certain species such as fungi where 18S rRNA and internal transcribed spacer sequencing represent a more appropriate target and viral identification requires approaches other than marker-gene-directed PCR analysis [24].

### Lung diseases

The effect of the lung microbiome composition on health and disease states is of interest not just in asthma research but for other respiratory diseases such as cystic fibrosis (CF), COPD, pulmonary fibrosis, and bronchiectasis. In bronchiectasis it is now believed that a change in composition of the lung microbiota through events such as antibiotic therapy and inflammation may have downstream consequences for the function of the immune system, allowing for the formation and preservation of chronic infection lead by microbial pathogens [25]. There is an appreciation that cystic fibrosis is a multisystem disease with involvement of the hepatobiliary and gastrointestinal tracts [26]. While gastro-oesophageal reflux (GOR) effects CF patients, the lung and gastric microbiomes and an inter-dependant relationship between the two is clearly defined. Gastric and sputum cultures have revealed that 73% of patients with CF had one or more microorganism (fungal or bacterial) in common between the two samples and among the bacteria found in the two sites were *Pseudomonas, Streptococcus* and *Achromobacter. Pseudomonas aeruginosa (Pa),* a bacteria typically associated with CF was found in the sputum samples of 11 patients of which 4 had identical *Pa* isolates in the stomach. In comparison, non CF gastric juice samples revealed a higher diversity. The stomach microbiome in patients with CF may act as a potential pathogen reservoir and the aerodigestive microbiome warrants further research as it may impinge on the pathophysiology of CF [27]. Bile acid aspiration is a comorbidity factor linked to many lung diseases which could plausibly shape the lung microbiome and promote the colonisation of *Pa* in CF patients. Using a systems based analysis involving transcriptomics, functional genomics and immunochemistry the network of genes involved in virulence, adaptive metabolism and antibiotic tolerance linked to the bile response of the pathogens and the host cells has been eludicated. Bile acid leakage into the oesophageal tract occurs in up to 80% of CF patients and occurs in up to 35% of COPD patients (although this figure is thought to be underestimated) [28]. Disease progression and antibiotic tolerance in respiratory patients may be linked to bile acid signalling. Bile acids can elict biofilm behaviour in *Pseudomonas aeruginosa*, increase polymyxin and macrolide antibiotic tolerance, and reduce microbial biodiversity [28]. COPD research is in its infancy with regards to the microbiota of the COPD lung however some recent studies have demonstrated that as the disease progresses there is a reduction in bacterial diversity however there is no demonstratable outgrowth of a single microorganism as is the case in CF where *Pseudomonas aeruginosa* predominates [29]. Patients with idiopathic pulmonary fibrosis (IPF) are reported to have a higher relative abundance of *Haemophilus, Streptococcus, Neisseria* and *Veillonella* species [30].

### Asthma characteristics

Asthma is a chronic inflammatory disease, which is characterised by airway hyper-responsiveness leading to intermittent, repeated bouts of wheezing, chest tightness, breathlessness and coughing [31, 32]. Asthma has a complex heterogeneity with many clinical phenotypes whose varying expression depends on interplay between numerous environmental factors along with many different susceptibility genes [1, 33]. The airway epithelium is pivotal in the inflammatory response of the host and is a major source of proinflammatory mediators [33]. T_H_2 cells subsequently play a prominent role in allergic disorder pathogenesis leading to B cells isotype switching, generating IgE antibodies which are specific to the particular insulting allergen [8]. T_H_2 cells further enlist and enhance survival of eosinophils and mast cells, induce goblet cell hyperplasia and further drive bronchial hyper reactivity [31]. The airway obstruction which characterises the clinical presentation of asthma runs in cycles of symptom free periods followed by varying periods of exacerbation to which there is usually a trigger or stimuli [34]. These exacerbations result in a worsening in symptoms and lung function and although is generally reversible and followed by a return to normal lung function, in some patients repeated exacerbations may lead to a new, compromised baseline [35, 36]. Such exacerbating triggers include infection, aeroallergens, pollution, drugs and physical stimuli [31, 37, 38]. These exacerbations are often treated with varying combinations of increased inhaled therapy, systemic corticosteroids and antibiotics [8]. One might postulate, therefore, that the exacerbating event and the mode of therapy utilised to treat the event may impact on the composition of the lung microbiome. Indeed recent studies have demonstrated that the gut microbiota can alter circadian rhythms which may open new avenues of research into identifying the cause of asthmatic exacerbations [39, 40]. It has been noted that an alteration to the *Bmal 1* (the clock gene) in bronchiolar cells disrupts the circadian neutrophil recruitment via CXCL5, which in turn leads to an exaggerated host response to LPS (lipopolysaccharide) and a deficient host immune response to *Streptococcus pneumoniae* [41].

### Epithelial development and the role of the microbiota

In the gastrointestinal tract there is a complex interplay between the symbiotic bacteria which are responsible for processes such as nutrient metabolism and the host which in turn provides a nutrient rich, protective environment. This interplay requires strict sequestration of the resident microbes and the host lumen to prevent the triggering of processes such as sepsis and inflammation. Epithelial antimicrobial proteins play a vital role in hindering the contact of resident microbes and the host mucosal surfaces. Germ-free mice and antibiotic treatment of murine models have demonstrated evidence that the microbiota is required for gut epithelia development and function. Cash et al.*,* first identified the role microbes play in inducing antimicrobial peptides and epithelial villus development and maturation [42]. It has been seen that in germ-free mice microbial colonisation induces the expression of RegIIIγ which is a secreted C-type lectin, known in humans as HIP/PAP. This expression directly correlates with bacterial expression and host epithelial contact, with preferential binding to peptidoglycan which is present in gram positive bacteria. This highlights how the host-microbial symbiotic relationship is maintained [42]. It is therefore possible that a similar protective mechanism could be involved in the lung mucosal epithelium.

Innate Lymphoid Cells (ILCs) have emerged as a population of innate immune cells derived from an Id2 dependent lymphoid cell progenitor cell population. ILCs share many functional and developmental characteristics with CD4^+^T cell populations. ILCs have been demonstrated to be involved in orchestrating certain host-commensal bacteria relationships [43]. Such an involvement would have implications on immunity, inflammation and tissue homeostasis in the intestines. In the lungs it has been seen that pathogenic ILC responses have been associated with asthma and airway hyperresponsiveness in murine models [43]. As commensal bacteria, epithelial cells and ILCs share close proximity and investigation into the potential impact of this interaction upon the development of ILCs is taking place. Hence ILCs may represent a potential target for numerous human diseases such as asthma [43].

Recent studies focusing on ILCs have also identified the key dialogue between ILCs, epithelia and the microbiota. IgA is the predominant antibody at mucosal surfaces, in particular in the upper respiratory tract. Through its pleiotropic effects IgA induces a tolerizing phenotype at mucosal surfaces and is of critical importance to the maintenance of mucosal homeostasis. IgA is created through the class switch recombination of Ig heavy chains. IgA offers an important role in mucosal homeostasis such as opsonisation and neutralisation of pathogens and toxins. This results in a reduction of antigenic load on the mucosal immune system. In IgA^−/−^ mice the protective role of IgA at mucosal surfaces is highlighted as these mice have an impaired clearance of luminal pathogens such as *Mycobacterium tuberculosis*, *Streptococcus pneumoniae*, *Citrobacter rodentium*, *Giardia muris* and *Giardia lamblia* [44]. It has been demonstrated that the microbiota acts through Toll-like receptors to condition lung dendritic cells (LDCs) to produce TGF-β and this in turn influences the production of IgA in the lung [44]. While research to date has focused mainly on the gut, evidence of bi-directional links between the gut and the lung immune function are increasing [45].

### Microbes and the host

A key feature of any microorganism is its ability to be recognised and sampled by the immune system. The ensuing immune response, depending on the microbe, can range from benign tolerance to acute inflammation and injury, with ensuing repair and remodelling in the lungs [3]. Respiratory fungi, bacteria and viruses are recognised by innate receptors including Toll-like receptors (TLRs), RIG-1-like helicases and NOD-like receptors which when stimulated then activate the nuclear factor κ B (NFκB) group transcription factors with the possible resultant induction of more than 100 pro-inflammatory and host response genes [3, 46]. Microorganisms can affect the permeability of the epithelium increasing infection risk, affecting epithelial integrity by invasion of the epithelium cells leading to cell death and shedding. By compromising the epithelium, subsequent allergen uptake and the effect of environmental exposure to pollutants may cause an increased possibility for the development of further exacerbations and a heightened immune response increasing the risk of a pathogenic response and asthma development [3, 47–49].

Asthma patients have an increased risk of bacterial infection and through culture dependent and independent techniques it has been seen that there is increased carriage of pathogenic respiratory bacteria. Birth cohort studies have shown that increased carriage of bacteria such as *Streptococcus pneumoniae, Haemophilus influenzae* and *Moraxella catarrhalis* in early life can be linked to asthma risk and the development of recurrent wheeze in children born to asthmatic mothers [50]. This study by Bisgaard et al.*,* also revealed that, in children with recurrent wheeze during the first 3 years of life, that these wheezing episodes are associated with bacterial and viral infections equally, suggesting that bacterial and viral infections may act independently to contribute to asthma symptoms and thereby highlighting the important role of bacterial infection independent of viral infection in the pathogenesis of asthma [51]. In a study by Hilty et al.*,* members of the phylum *Proteobacteria* were associated with airway disease in both asthma and COPD patients. *Haemophilus*, *Moraxella* and *Neisseria* spp. found in the lungs of asthmatic patients had strong correlations with increased asthma risk when found in the oropharynx of neonates. *Staphylococcus* was found to be present in the airways of children with difficult asthma. *Bacteroidetes* and in particular *Prevotella* and *Veillonella* spp. were found to be most prevalent in healthy controls [2].

While microorganisms can add to the onset and progression of atopic asthma, microorganisms can also have a protective function [3]. The ‘hygiene hypothesis’ can in part help to explain the disproportionate spread of asthma in the western, industrialised world. It proposed that repeated exposure to diverse common infections and repeated exposure to environmental microbes during childhood can stimulate the immune system in a manner which promotes a protective responseagainst future asthma risk [3].

The so-called ‘hygiene hypothesis’ contends that exposure to soil, dust, microbes, antibiotics, vaccinations, farm animals and family size in addition to factors such as caesarean section birth versus vaginal birth and exclusive formula feeding versus breastfeeding can all in some part determine asthma risk [52–54]. In one meta-analysis study there was a 20% increase in the subsequent risk of asthma development in children born by caesarean section [55].

The use of anti-inflammatory drugs and antibiotics can significantly alter the lung microbiome [56]. Such a change in the microbiome of the lung may lead to resistance, resilience, functional redundancy, or a permanently altered microbiome [11]. It is seen that even before birth children are exposed to antibiotics, with about half of all pregnant women in America receiving antibiotics which alter their microbiota before transfer to their offspring [11].

Environmental microbial exposure can have a protective effect against asthma development. In the PARSIFAL and GABRIELA studies asthma and atopy prevalence among children living on farms and among children in the reference group (not living on a farm) were examined. In both studies children living on a farm had a lower prevalence of asthma than the reference group From these two large scale observational studies it would appear that a wider range of microbial exposures offers a protective effect on the development of asthma [57].

It has been shown that single nucleotide polymorphisms (SNPs) in TNFAIP3 which encodes A20 is linked to the development of asthma in farm dwelling children. A20 is an ubiquitin-modifying enzyme which weakens NFκB activation through the deubiquitination of key signalling intermediates down-stream of IL-1 receptor, Toll-like receptor and TNF family receptors. It is accepted that chronic exposure to low-dose endotoxin or farm dust conveys a protective effect against the development of house dust mite (HDM) induced asthma in mice and loss of A20 is associated with allergy and asthma risk. It has been shown that in mice with an induced absence of A20 there is an increased sensitivity to HDM and the ensuing asthma is seen to be more severe [58].

Children from rural areas in Germany, Austria and Switzerland from farming and non-farming households were involved in a study to determine whether there was a protective effect present upon endotoxin exposure against the risk of asthma and atopic wheeze. Mattress dust, as an indicator for endotoxin exposure, was examined and it was found that environmental exposure to endotoxins was associated with the development of tolerance towards ubiquitous allergens found in natural environments [59]. Muramic acid, a component of bacterial peptidoglycan, is a marker for microbial burden and is a biologically active substance which influences the cellular immune response in a manner different to endotoxin. A high level of muramic acid in a similarly constructed study provided complimentary findings, lending further credence to the notion that asthma could in part be due to inadequate activation, at the appropriate educational time point, of the innate immune responses which are stimulated by certain microbial products [60].

Recent studies, including the Canadian CHILD cohort study, have highlighted the importance of this window of colonisation in children and long-term side effects such as asthma which can ensue when there is a presence or lack of certain microorganisms in the gastrointestinal system. It was determined that the relative abundance of the bacterial genera *Faecalibacterium, Lachnospira, Veillonella,* and *Rothia (*FLVR) appeared notably decreased in the gut of children who were at risk of developing asthma [61]. These microorganisms appeared to be mostly significantly different between the groups at 3 months and this difference decreased once the children reached 1 year of age, suggesting the presence of a colonisation “window of opportunity” [61, 62]. It was also seen that it is not just the prevalence of the gut microbiota that is important but the metabolites that they derive, such as the short chain fatty acids (SCFAs) propionate and acetate which have been demonstrated in a murine model to reduce airway cellular infiltration [61, 63]. This concept of a “window of opportunity” for colonisation, while now generally accepted, requires further investigation. In mice this critical window occurs during the first 2–3 weeks of life and it is expected that in humans the critical window is narrower than first expected and rather than years is likely to merely last for a matter of months [62].

In germ-free mice it has been shown by Olszak et al., that there is dysregulation in the development of an immune cell profile when there has been no microbial exposure. However if colonised gastrointestinally during a crucial neonatal period with a microbiome, a protective effect against exaggerated inflammation developed and this effect could not be demonstrated in recolonised adult mice [64]. Hence, one might postulate that if microbial exposure can alleviate or prevent the immune cascade as seen with allergic disorders such as asthma, could a prebiotic or probiotic have a protective or even preventive role in asthma? It has been seen that *Lactobacillus reuteri*, when administered to mice demonstrating a normal microbial flora, can abate airway hyperresponsiveness via systemic increase in regulatory T cells, which can therefore alleviate asthma symptoms [65–67]. It was seen also that direct exposure to an innocuous strain of *Escherichia coli* could result in long term protection against allergic disorders in normal mice by reprogramming macrophages and dendritic cells [68]. Dendritic cells bridge the gap between innate and adaptive immunity [52]. In BALB/c ovalbumin challenged mice fed daily with *Bifidobacterium longum* it was noted that airway inflammation was attenuated and a reduction in BAL eosinophilia, BAL fluid IL-4 protein and BAL cell IFN-γ and IL-4 mRNA levels was observed [69]. To test the effect of early life antibiotic exposure to the microbiota and subsequent allergic asthma susceptibility Russell et al.*,* treated neonatal mice with clinical doses of Vancomycin and Streptomycin. Changes in the resident gut microbiota and subsequent asthma susceptibility were documented. In mice treated with Streptomycin there was no significant effect upon the microbiota or asthma risk. However in mice treated with vancomycin it was noted that there was a reduction in microbial diversity and changes in bacterial population composition were observed leading to a heightened risk of asthma development upon allergic challenge. As neither antibiotic had an effect upon adult mice, one might infer that the hygiene hypothesis is of relevance only in neonatal shift in microbial diversity and composition [70]. Russell et al., investigated whether or not this effect held true for perinatal antibiotic treated mice and the risk of developing hypersensitivity pneumonitis, a T_H_1–T_H_17 mediated lung disease. Contrary to the previous findings severity of the disease increased dramatically with Streptomycin. It was concluded that perinatal antibiotics affect the resident gut microbiota in a highly selective manner and this then leads to a change in susceptibility to T_H_2 or T_H_1-T_H_17 driven lung inflammatory diseases [71].

The prevalence of *Helicobacter pylori,* which was once the dominant bacteria in the stomach and had strong maternal transmission, is now decreasing and with it possibly the protective effects this bacteria seems to have against asthma development [72]. Studies have shown that childhood acquisition of *H. pylori* reduces the risk of allergy and asthma [72]. In developing countries nearly all adults harbour the gastric bacterium *H. pylori* but in industrialised countries its prevalence is much lower [73]. In industrialised countries such as the United States fewer than 10% of children under 10 years of age are colonised with *H. pylori* [74]. However it should be noted that *H. pylori* acquisition does not appear to be associated with adult onset asthma suggesting that the pathogenic risk factors for adult onset asthma may differ from those for childhood asthma [74].

It is believed that microaspiration, which occurs in healthy individuals but has a higher prevalence in asthmatics, could in part explain the presence of oral microbiota in the lower lung [15]. However this does not fully explain why some individuals have a higher relative abundance of certain bacteria originating from the upper respiratory tract [15, 75].

In a recent study by Marri et al.*,* induced sputum samples from a mild active asthmatic and non-asthmatic cohort of adults was examined and it was determined that all sputum samples contained the 5 main bacterial genera with *Firmicutes, Proteobacteria* and *Actinobacteria* making up 90% of the sequence reads. It was further shown that *Proteobacteria* levels were elevated in asthmatics and *Firmicutes* and *Actinobacteria* were more common in the non-asthmatic cohort. From this study it was determined that mild asthma was more similar in microbial composition to that of severe asthmatics that to the non-asthmatic group [76].

The “disappearing microbiota” hypothesis contends that as we become less colonised by ancient commensal microorganisms, which aid in a multitude of processes such as vitamin uptake and immunity, we become more susceptible to attack by potential pathogenic microorganisms. A shift in an individual’s microbial balance may have detrimental consequences. The “disappearing microbiota” hypothesis has been studied in relation to the increased and decreased prevalence of several diseases in developed nations. While the hygiene hypothesis highlights our susceptibility to infection caused by our decreased sampling of microorganisms from the environment the disappearing microbiota hypothesis suggests that our susceptibility is due to a decrease in ancestral microorganisms which offered protection [77].

### Lung epithelia and mucosal immune defences

While the role of the gut microbiota in shaping the mucosal immune system, the symbiosis between the gut microbiota and intestinal mucosa, and the importance of barrier function within the gut is well understood, there remains a paucity of information pertaining to the lung [11]. As the lungs are directly exposed to the environment the epithelium is the first line of defence against infection [78, 79]. In fact the lungs have the largest epithelial surface of the body [80]. By producing inflammatory products such as cytokines, growth factors, chemokines, lipid mediators, peptide mediators and reactive oxygen/nitrogen species the respiratory epithelial cells regulate the inflammatory response in the airways [81]. The epithelium acts as a barrier which prevents inhaled pathogens gaining access. Club and type II alveolar epithelial cells produce a surfactant which effectively traps pathogens and mucociliary clearance aid pathogen removal. The epithelium can function as a catabolic barrier for proteases and peptides with the epithelium expressing cell surface peptidases and producing protease inhibitors. The epithelium barrier function is further augmented by integral tight junction proteins [81]. The cells of the innate immune response include macrophages, neutrophils and monocytes which utilise pathogen recognition molecules (PRMs) to recognise pathogen association molecular patterns (PAMPs) leading to the production of antimicrobial products [78, 81]. Well known cell surface receptors include Toll-like receptors which can recognise a wide variety of viral and bacterial PAMPs such as LPS [78, 82].

Asthma is a heterogenous disease, with airway inflammation induced by epithelial exposure to allergens, pollutants and microbes which further stimulate the underlying APC (antigen presenting cells) cells inducing an immune response [83]. This epithelial exposure can result in a predominantly eosinophilic or neutrophilic cell influx into the airway. The allergic subtype, which responds to environmetal allergens, is characterised by increased eosinophil, basophils, and mast cells along with expansion of the T helper 2 cell (T_H_2 cell) subtype. As the epithelial or mucosal barrier is where the allergen presents itself, a breakdown in these physical barriers, the site of allergen presentation, can further propagate allergic, inflammatory response [8]. Indeed fungal spores, which are potent allergens, are postulated to damage the epithelial barrier by inducing cell shrinkage and subsequent inflammation and barrier breakdown [84].

The interaction of the APC with the epithelium is the critical event in the resulting immune response. Dendritic cells act as sentinels, reacting to the exposure to certain allergens and pathogens from the environment. Dendritic cells activate an appropriate immune response after exposure to an allergen dependent on the appropriate mechanism of action for clearing the invading pathogen. It has been noted that mice lacking in DCs have major deficiencies in their antibacterial, antiviral and antifungal immunity. From recent murine studies it is believed that the epithelium has a key role in the activation of dendritic cells after exposure to an adverse pathogen or allergen and that there is an established epithelia-dendritic cell communication pathway [85].

Alveolar macrophages are the primary microbial sampling and clearance mechanism within the lung lumen and play an integral role in pulmonary host defense positioned at the interface between the mucosa and the external environment [86]. The composition of the microbiome in an asthmatic lung will consequentially effect macrophage sampling. Indeed the function of the alveolar macrophages may also effect what microbes persist in the lung and hence influence the resulting airway microbiome. Studies on alveolar macrophages in smokers have demonstrated that accumulation of tobacco smoke particulates in the lysosome effects migration and response to *Mycobacterium tuberculosis* [87]. These observations should also be noted with regard to asthmatics and their lung microbiota, as failure to effeciently clear certain species may result in persistence and imbalance.

### The Virome and asthma

Epidemiological studies across five continents spanning three decades have revealed a strong link between respiratory infections and the pathogenesis of asthma [3]. Respiratory syncytial virus (RSV), metapneumovirus, coronavirus, parainfluenza and human rhinovirus are just some of a plethora of human viruses which are known to be connected with respiratory diseases like asthma [88]. Human rhinoviruses can act as a trigger for asthma exacerbations [81]. Viral respiratory tract infections, most commonly rhinovirus infections, can have a serious effect on asthmatic patients or people at risk of developing asthma, causing exacerbations and worsening disease prognosis [88]. Some emerging evidence has shown that individuals with asthma may have deficiencies in antiviral activity and a dysfunctional epithelial barrier, increasing susceptibity to severe viral respiratory infections with more potential for exacerbations [88]. Sigurs et al.*,* have determined that acute RSV infections, occurring in conjuction with a family history of asthma, synergistically increase the risk of asthma development in an invidual, illustrating that this gene-by-environment interaction has prognostic implications in asthma [88, 89]. Viruses have been seen to predispose individuals to secondary bacterial infection. Upon exposure to RSV there is an increase in the adherence of *Streptococcus pneumoniae* to human epithelial cells [81, 90]. Respiratory virus infection markedly increases emergency room admissions in relation to asthma exacerbations [3]. Wheezing respiratory viral illnesses from birth to 3 years of age, in high risk children (parent who tests positive for respiratory allergies or a history of diagnosed asthma) increase the risk of asthma development 10 fold when the child reaches 6 years of age [91]. It has also been shown that severe respiratory syncytial virus bronchiolitis in infancy, predisposes to the development of adult asthma [92]. Respiratory virus (RV) and RSV are capable of activating a range of proinflammatory cytokines, chemokines, growth factors, adhesion molecules and mucins, relevant to the pathophysiology of asthma [3].

### The mycobiome and asthma

The bacterial microbiome of the lungs has been the main focus of research to date, however the fungal microbiome/mycobiome of the lungs warrants investigation [93]. A marker for fungal exposure, extracellular polysaccharides, derived from *Aspergillus* and *Penicillium* are now known to be inversely associated with asthma [94]. A recent investigation of pooled sputum samples from asthma patients and controls identified 136 fungal species of which 90 species were more common in asthmatics. *Psathyrella candolleana, Malassezia pachydermatis, Termitomyces clypeatus* and *Grifola sordulenta* were most commonly found in asthmatics and *Eremothecium sinecaudum, Systenostrema alba, Cladosporium cladosporioides* and *Vanderwaltozyma polyspora* were more prevalent in the sputum samples of the control group [95]. Wheeler et al.*,* have demonstrated that antifungal drugs cause a fungal dysbiosis in mice and this dysbiosis results in an increase in the severity of airway inflammatory diseases such as asthma [96]. It has been well recognised for centuries that mould exposure can lead to an exacerbation of asthma [84]. In certain patients, mould exposure can have detrimental effectsleading to increased disease severity, hospital admissions, and increased risk of bronchial reactivity and death [84]. Environmental risk factors such as pollen, animal dander and mould spores and their impact on asthma were examined from 1985 to 1989 in a cohort of asthmatics ranging in age from 5 to 34 years in Chicago, US. It was discovered that there was a 2.16 times greater chance of an asthma related death on days when the mould spore count reached >1000 spores per cubic meter. While factors such as genetics and the patients socioeconomic situation have important roles, there appears to be a temporal relationship between the high environmental spore count and subsequent asthma attacks [97]. The summer-autumn period of asthma admissions and deaths corresponds with the peak in environmental mould spores seen at this time [84].

It has been reported by Black et al.*,* in 37 patients admitted to the ICU for an acute asthma attack, that 54% tested positive in a skin test for fungal spores which included *Alternaria tenuis, Cladosporium cladosporides, Epicoccum nigrum and Helminthosporium maydis* [98]*.* O’ Driscoll et al.*,* recruited 181 patients, aged between 16 and 60 years of age and divided them into three groups; severe, moderate and mild, dependent on their number of lifetime hospital admissions. 76% of patients with severe asthma tested positive in a skin test toone or more moulds including *Aspergillus fumigatus, Penicillium notatum, Cladosporium herbarum, Alternaria alternata* or the yeast *Candida albicans* in comparison to lower rates of positivity in patients with moderate or mild asthma (16–19%) [99].

Exposure to fungal allergens can have a devastating impact onasthmatics. Fungi contain proteins which are detrimental to the airway epithelium, enhance additional reactions and also act as allergens [84]. It is possible that the long term fungal colonisation of an atopic patient may provide a chronic source of allergen exposure, propagate airway inflammation and increase severity of asthma phenotype [84].

## Conclusion

There is an emerging consensus that the microbiome potentially plays a critical role in disease development, including in the pathogenesis of airway conditions such as asthma [100]. Asthma rates are increasing and thanks to meta-genomics techniques it is now possible to define the composition of microbes in the lungs. It is now possible to determine whether changes in the composition of the lower airway microbiota influences the complex pathophysiology of a chronic lung disease such as asthma. Emerging studies have demonstrated that the role of the gut and lung microbiota and the resulting immune engagement cannot be ignored when studying asthma disease progression. Recent studies have demonstrated that oral antibiotics and antimycotics alter the gut microbiota exacerbating allergic asthma symptoms in mice. The resulting reduction in gut microbial diversity and modifed population composition was found to lead to increased allergic symptoms. Modification of commensal microbial species such as clostridia species have been implicated in these observations. Indeed clostridial dervied SCFAs, such as butyrate and actetate, are responsible for inducing mucosal Treg differentation and function [63, 101]. Studies to date suggest that a disrupted microbiota or shift in an individual’s microbial balance could possibly have detrimental effects. Further research is now required to fully dissect this role and ultimately pave the way for the emergence of new therapeutic strategies in combating these conditions.

## Abbreviations

APC: Antigen presenting cell
BAL: Bronchoalveolar lavage
CF: Cystic fibrosis
COPD: Chronic obstructive pulmonary disease
GOR: Gastro-oesophageal reflux
HDM: House dust mite
HIP/PAP: Hepatocarcinoma-intestine-pancreas/pancreatic associated protein
ICLs: Innate lymphoid cells
LDCs: Lung dendritic cells
LPS: Lipopolysaccharide
Pa: Pseudomonas aeruginosa
PAMPs: Pathogen associated molecular patterns
PRMs: Pathogen recognition molecules
RSV: Respiratory syncytial virus
RV: Respiratory virus
SCFAs: Short chain fatty acids
SNPs: Single nucleotide polymorphisms
T_H_1: T helper 1
T_H_2: T helper 2
TLRs: Toll-like receptors
